# Annual Commencement of the Buffalo Medical College

**Published:** 1859-03

**Authors:** 


*Annual Commencement of the Buffalo Medical College.*—This institution closed its session on the 23d ult., with a much larger class than usual. The number of students in attendance was sixty-seven, thirteen of whom received the degree of Doctor of Medicine. The commencement exercises took place at American Hall in this city, when the degrees were conferred by the Hon. Millard Fillmore, Chancellor of the University, who made a few appropriate remarks. Prof. Hamilton then followed in an able charge to the graduates, in which he took up, among other things, the subject of hygiene and its bearings upon our natural physique. Seldom have these interesting exercises been witnessed by a larger or a better audience; the interest manifested by our citizens was alike flattering to the speaker and the institution which he represented.

